# Physico-chemical, sensory, and microbiological quality of raw chicken meat: an exploratory study in the Hisar city of Haryana, India

**DOI:** 10.3389/fnut.2023.1184005

**Published:** 2023-07-18

**Authors:** Indora Bhawana, Ashok Malik, António Raposo, Shubha Singh, Sanjay Yadav, Renata Puppin Zandonadi, Linda Heejung Lho, Heesup Han, Neha Thakur

**Affiliations:** ^1^Department of Livestock Products Technology, College of Veterinary Sciences, Lala Lajpat Rai University of Veterinary and Animal Sciences, Hisar, Haryana, India; ^2^CBIOS (Research Center for Biosciences and Health Technologies), Universidade Lusófona de Humanidades e Tecnologias, Lisboa, Portugal; ^3^University of Brasília, Faculty of Health Sciences, Nutrition Department, Campus Universitário Darcy Ribeiro, Brasília, Brazil; ^4^College of Business Division of Tourism and Hotel Management, Cheongju University, Cheongju-si, South Korea; ^5^College of Hospitality and Tourism Management, Sejong University, Seoul, South Korea

**Keywords:** raw chicken meat, microbial quality, sensory evaluation, physico-chemcial evaluation, meat hygiene

## Abstract

A survey was conducted in Hisar, located in Haryana, India, to assess the quality of raw chicken meat. To ensure comprehensive coverage, healthy broiler chickens were obtained from various meat retail outlets in Hisar city, encompassing the majority of such establishments. Additionally, a sample of control chickens was obtained from the Livestock Farm, College of Veterinary Sciences, Lala Lajpat Rai University of Veterinary and Animal Sciences (LUVAS), Hisar, Haryana, India. The raw chicken meat was grouped into two categories, breast cut and thigh cut. The breast muscles, which include *pectoralis major* and *pectoralis minor*, and the thigh muscles, which include the abductor muscles, were chosen as the samples for proximate analysis, which included physico-chemical, sensory, and microbiological analyses of raw chicken meat. The analysis of the raw meat in the laboratory revealed inconsistent variations between the control and retail samples in terms of parameters, such as proximate composition, pH, the water-holding capacity (WHC), thiobarbituric acid (TBA), instrumental color analysis, and sensory evaluation. The moisture content of the control breast sample was significantly higher (*p* < 0.05) than that of the samples from shops 2, 3, and 5. However, it was statistically similar to that of the samples from shops 1, 4, and 6. The total plate and psychrotrophic counts of the control thigh sample were significantly lower than those of the samples from shops 3, 4, 5, and 6. Among the thigh pH values, the samples from shops 1, 2, 5, and 6 had significantly higher pH values than the control sample. The variations in the various parameters were multifactorial and established the superiority of birds slaughtered under laboratory conditions and grown in university farms compared to the raw chicken meat available in retail outlets in Hisar city.

## 1. Introduction

Meat is regarded as a valuable food from a nutritional standpoint, and it is one of the main components of the daily diet of a significant percentage of humans. Since the dawn of human civilization, agriculture has played a significant role for humankind (1). Animal husbandry, dairying, and other activities related to livestock rearing are all part of agriculture. These endeavors ensure food security, promote independence, and uphold ecological balance, which are essential to sustainable development. Humans were hunters and gatherers before they cultivated crops and animals, and they were influenced by traditional, cultural, and religious beliefs (2).

Muscle meal proteins, which include a net protein utilization value of approximately 0.75 compared to 0.5–0.6 for plant protein, have high bioavailability (3). The majority of Hindus in India are opposed to beef consumption, whereas pork consumption is forbidden among Muslims. Religious reasons, conventions, and taboos govern meat intake in India. Consumers in India are far more accepting of the consumption of chicken meat compared to other types of meat (4).

The demand for meat differs with respect to quantitative and qualitative parameters due to the shifting consumer tastes and preferences for meat (5). Chicken is the most popular meat in India, but the demand for it varies significantly depending on economic status, family values, holidays, and considerations for animal welfare. India is the world's fourth-largest producer of broilers, and the country produces an estimated 4.1 metric tons (MT) of broilers yearly (6). Slaughtering, preparing, and processing poultry exposes the meat to microbial contamination from various sources. The immediate initiation of lipid oxidation and the high microbial load of poultry meat result in low sensory ratings and short storage times for products produced with poultry meat (7). Subpar hygiene and sanitation procedures among bird meat suppliers can lead to meat contamination (8).

A meat hygienist's primary responsibility is to ensure that customers are protected from foodborne illnesses. Microbiological food safety is currently viewed as a problem for developing societies. *E. coli, Salmonella*, and *Staphylococcus aureus* infections are extremely common in undeveloped communities, and they pose a serious threat to human health (9).

The Hazard Analysis and Critical Control Points (HACCP) system should be closely adhered to control foodborne illnesses and limit the microbial load of raw meat. However, the degree of sanitation, the methods of transportation, and the circumstances of storage all contribute to meat contamination and the proliferation of many dangerous types of bacteria in developing nations, such as India. Foodborne diseases are increasing in Hisar city, India, where the hygiene of raw chickens sold by retail establishments is questionable. The area is also overcrowded and has poor sanitary conditions (10). This study aimed to evaluate and compare the quality of raw chicken meat—produced on a university farm and slaughtered in a departmental abattoir—with market meat—procured from different retail outlets/vendors.

## 2. Materials and methods

The present investigation was conducted in the Department of Livestock Products Technology, College of Veterinary Sciences, LUVAS, Hisar, Haryana, India.

### 2.1. Sample collection

Healthy broiler chickens that were slaughtered and dressed in six meat retail outlets in Hisar city were obtained. The details of the shops are mentioned in Appendix I. The control sample, which was hygienically slaughtered and dressed as per the standard procedure in the Meat and Meat Processing Laboratory, Department of Livestock Products Technology, Lala Lajpat Rai University of Veterinary and Animal Sciences, Hisar, Haryana, India, was obtained from the Livestock Farm, College of Veterinary Sciences, Lala Lajpat Rai University of Veterinary and Animal Sciences, Hisar, Haryana, India. The raw chicken meat was grouped into two categories, breast cut and thigh cut. The breast muscles, which include *pectoralis major* and *pectoralis minor*, and the thigh muscles, which include the abductor muscles, were chosen as the samples for further analysis, which included physico-chemical, sensory, and microbiological analyses of raw chicken meat.

### 2.2. Quality evaluation

Raw chicken meat samples were collected from six retail outlets and one university farm to conduct physico-chemical, sensory, and microbiological analyses. The standard methods of Association of Official Agricultural Chemists (AOAC) were followed to determine the proximate composition (1995). The determination of pH (11), TBA (12), the water-holding capacity (13), and instrumental color analysis (Konica Minolta chroma meter CR-400, Konica Minolta Sensing, Inc., Japan) were undertaken in the departmental laboratory. Additionally, sensory and microbial quality (14) evaluations were performed alongside the given parameters.

#### 2.2.1. Proximate composition

The standard methods of AOAC (15) were followed to determine the proximate composition.

a) Moisture

The chopped sample (30 g) was weighed in a dry aluminum dish and placed in a hot air oven, where it was exposed to 100–105°C for 16–18 h with the lid open. The weight loss was calculated by measuring the moisture content of the sample after it had cooled in a desiccator.

b) Crude protein

Reagents:

(i) H_2_SO_4_ (conc.)
(ii) H_2_SO_4_ (0.1 N)
(iii) Boric acid solution (4 %)
(iv) NaOH solution (40 %)
(v) Catalyst: Copper sulfate and potassium sulfate (1:5)
(vi) Mixed indicator: 0.3 g of bromocresol green and 0.2 g of methyl red were dissolved in 400 ml of 90% ethanol and stored in a dark brown bottle.

Procedure

A semiautomatic instrument by Pelican Equipment (Chennai, Tamil Nadu, India) was used to conduct protein estimation. In particular, 0.25 g of the moisture-free sample and 10 ml of conc. H_2_SO_4_ was transferred to a Kjeldahl digestion tube. A pinch of the catalytic mixture was added, and digestion was conducted until a blue-green clear solution was obtained. The aliquot was diluted with distilled water after it had cooled. The diluted aliquot was made alkaline by mixing it with a 40% NaOH solution and distilled water. Liberated ammonia was collected in a conical flask that contained 25 ml of boric acid solution and 2–3 drops of the mixed indicator. The contents of the flask that contained boric acid were titrated against 0.1 N H_2_SO_4_.


$$\begin{array}{l} \text{Protein}(\%)=\\ \frac{\text{14 }\times \text{ normality of acid used }\times \text{ volume of acid used}\times \text{6}.\text{25}\times \text{100}}{\text{Weight of sample }(\text{g})\times \text{1000}} \end{array}$$

c) Ether extract

The fat content in the cooked product was estimated by using the solvent extraction method (15) employing Socs Plus (SCS-6-AS, Pelican Industries, Chennai, Tamil Nadu, India). A total of 2 g of the ground and dried samples were placed in an extraction thimble. The initial weights of the empty beakers were noted. The thimbles with the samples were placed in weighed beakers that contained approximately 80 ml of the solvent (petroleum ether). Fat extraction was conducted automatically using the three part programs. The beakers were removed after fat extraction and dried in a hot air oven at approximately 105 ± 5°C for 1 h, and they were cooled in a desiccator and weighed. The weight gained by the beakers was calculated as the fat of the samples.

d) Ash

A total of 2 g of the moisture-free sample was placed in a dried and pre-weighed silica crucible. It was heated on a hot plate until the smoking ceased, and the samples became thoroughly charred. The charred samples were then kept in a muffle furnace at 550 ± 5°C for 2 h. The crucible was taken out after the furnace cooled, cooled in a desiccator, and the final weight was recorded. The ash content was determined by calculating the difference between the weight of the empty crucible and the weight after ashing.

#### 2.2.2. pH

The method by Trout et al. (11) was followed to determine the pH of the meat samples. The meat samples (10 g) were blended with 50 ml of distilled water for 1 min using a pestle and mortar. The pH was recorded by dipping the glass electrodes of the pH meter (Cyber Scan pH 510, Eutech Instruments; Thermo Fisher Scientific, Navi Mumbai, Maharashtra, India) directly in the suspension.

#### 2.2.3. TBA

The TBA value was determined according to the method by Witte et al. (12).

Reagents

(i) Tetra ethoxy propane (TEP) stock solution (0.31 ml) was diluted to 100 ml with
(ii) 95% ethyl alcohol. The strength of the solution was 3 mg/ml.
(iii) The TEP (working standard solution) stock solution (0.1 ml) was diluted to 100 ml with distilled water. The strength of the solution was 3 μg/ml.
(iv) Thiobarbituric acid reagent (1 mg/ml)
(v) Trichloroacetic acid (20%)

Extraction

Minced meat (5 g) was blended for 3 min with 25 ml of 20% TCA. The slurry was kept for 10 min. It was filtered through a Whatman No. 42 filter paper. A total of 5 ml of the TBA reagent was added to 5 ml of the sample aliquot (filtrate). The tubes were kept in a boiling water bath for 35 min after mixing the contents. The optical density was spectrophotometrically measured at 532 nm.

#### 2.2.4. WHC

The WHC was estimated based on the methods by Wardlaw et al. (13) with slight modifications. The finely minced meat sample (20 g) was placed in a 100-ml polycarbonate centrifuge tube, and then, 30 ml of the 0.6-M NaCl solution was added to it to prepare a meat slurry. Later, the meat slurry was mixed with a glass rod and stirred for 2 min using a mechanical shaker. It was again stirred for 1 min using a shaker and immediately centrifuged at 5,000 rpm for 15 min at the refrigerated centrifuge (Eltek refrigerated centrifuge, Model MP 400R) after holding it for 15 min at 4°C to allow the effect of the salt to reach equilibrium. The supernatant volume was measured, and the difference between the added and the decanted solution was expressed as a percentage of the initial weight of the meat sample.

#### 2.2.5. Instrumental color analysis

The color of the samples was measured using a Konica Minolta chromameter CR-400 (Konica Minolta Sensing, Inc., Japan) with an 8-mm aperture for the measurement. The instrument was calibrated with a white standard plate. The color scores were expressed as CIE Lab L^*^ (lightness), a^*^ (redness), and b^*^ (yellowness).

#### 2.2.6. Sensory evaluation

An experienced panel of five judges, who were faculty members from the department, evaluated the samples for sensory attributes, such as color, aroma, general appearance, and overall acceptability, using a 5-point descriptive scale. The test samples were presented to the panelists after suitable codes were assigned to them. A tissue paper was used to clean the hand between the samples. Appendix – II presents the sensory evaluation form used in this study.

#### 2.2.7. Microbiological quality

The total plate, total coliform, and psychrotrophic counts of the samples were enumerated by following the methods described by FSSAI (14).

##### 2.2.7.1. Preparation of the sample and the serial dilutions

The samples were opened in an inoculation chamber of laminar flow (RH-58-03, Rescholar Equipment, Ambala, India) pre-sterilized using ultraviolet (UV) radiation. A total of 10 g of the opened samples were aseptically weighed and transferred to a pre-sterilized mortar that contained 90 ml of sterile 0.1% peptone water (RM001; Hi-Media Laboratories Pvt. Ltd., Mumbai).

The sample was homogenized for 2 min using a sterile pestle and mortar to ensure uniform dispersion and to obtain a 10^−1^ dilution of the sample. A total of 1 ml of this diluted sample was quantitatively transferred and mixed to uniformly prepare a 10^−2^ dilution in a test tube that contained 9 ml of sterile 0.1% peptone water. Then, 1 ml of the 10–2 dilution was again added to 9 ml of sterile 0.1% peptone water and mixed to obtain a 10–3 dilution. The sample preparation and serial dilutions were conducted near a flame in a horizontal laminar flow apparatus, and all possible aseptic conditions were observed. The serial dilutions were made as per the requirements.

##### 2.2.7.2. The total plate count

A total of 23.5 g of plate count agar (M091; Hi-Media Laboratories Pvt. Ltd., Mumbai, Maharashtra, India) was suspended in a 1,000 ml glass of distilled water and boiled to completely dissolve the medium. The glass was sterilized through autoclaving at a pressure of 15 lbs and at a temperature of 121°C for 15 min. The final pH of the medium was set at 7.0 ± 0.2 at 25°C. The pour plate technique was used. The plates were incubated at 35°C for 24–48 h in an inverted position.

Plates with 30–300 colonies were manually counted, and the average number of colonies was multiplied by the reciprocal of the dilution and expressed as log_10_ cfu/g of the sample.

##### 2.2.7.3. Total coliform count

A total of 41.5 g of violet red bile glucose agar was procured from Hi-Media Laboratories Pvt. Ltd., Mumbai (ME581). It was suspended in 1,000 ml of distilled water, boiled to dissolve the medium completely, and cooled to 45°C. The final pH of the medium was adjusted to 7.4 ± 0.2 at 25°C. A total of 1 ml of suitable dilution in duplicate was pipetted into a sterilized petri dish. Approximately 20 ml of the melted medium was poured over it, and it was slowly mixed with rotating actions. The plates were allowed to stand for some time until the agar media solidified. Then, another 4-5 ml of agar was added to form an anaerobic layer after solidification, and the agar medium was allowed to solidify. The plates were incubated at 35 ± 2°C for 24 h. The numbers of red-purple colonies with approximately 0.5 mm diameter surrounded by a zone of precipitated bile were counted. The colonies that were judged to be borderline cases were also counted. The average number of colonies was multiplied by the reciprocal of the dilution and expressed as log cfu/g.

##### 2.2.7.4. Psychrotrophic count

The media and protocol of enumerating psychrotrophic counts were the same as those of the Total Plate Count (TPC), except for the temperature and the time of incubation. The plates were incubated at 4 ± 1°C for 5–7 days in an inverted position.

### 2.3. Statistical analysis

The obtained data were statistically analyzed by performing the Duncan test with SPSS-16.0 (SPSS Inc., Chicago, II USA) software to determine the significant difference at a 5% significance level for the mean values.

## 3. Results

### 3.1. Proximate composition of chicken meat

#### 3.1.1. Proximate composition of chicken breast

The moisture content of the control breast sample was significantly higher (*p* < 0.05) than that of the samples from shops 2, 3, and 5. However, it was statistically significant to that of the samples from shops 1, 4, and 6. The protein content of the breast samples from the control and the retail shops ranged from 20.87% to 21.61%. The protein percentage of the breast sample from shop 2 was the highest, and it was statistically comparable to that of the control sample and those from shops 1, 3, 5, and 6. The protein percentage of the breast sample from shop 4 was the lowest among all the shops. The fat content of the control sample and that from shop 3 was significantly higher (*p* < 0.05) than the samples from other shops. The ash content of the control was significantly higher (*p* < 0.05) than that of the samples from shops 4 and 6, and it was statistically comparable to the ash content of the samples from shops 1, 2, 3, and 5, as presented in Table 1.

**Table 1:** Proximate composition of the raw chicken breast and thigh muscle samples obtained from the Livestock Farm, COVS and six different retail outlets across Hisar, Haryana, India (Mean ± SD) (*n* = 6).

**Proximate composition**	**Moisture (%)**	**Protein (%)**	**Fat (%)**	**Ash (%)**
**Control (BREAST)**	74.13^a^ ± 0.59	21.59^ab^ ± 0.77	2.57^a^ ± 0.26	1.49^a^ ± 0.25
**SHOP 1 (BREAST)**	73.92^ab^ ± 0.53	21.54^ab^ ± 0.74	2.09^b^ ± 0.32	1.44^ab^ ± 0.32
**SHOP 2 (BREAST)**	73.33^b^ ± 0.84	21.61^a^ ± 0.49	2.20^b^ ± 0.28	1.29^ab^ ± 0.19
**SHOP 3 (BREAST)**	73.32^b^ ± 0.78	21.56^ab^ ± 0.75	2.51^a^ ± 0.40	1.36^ab^ ± 0.34
**SHOP 4 (BREAST)**	73.48^ab^ ± 0.91	20.87^b^ ± 0.89	2.23^b^ ± 0.29	1.21^b^ ± 0.31
**SHOP 5 (BREAST)**	73.36^b^ ± 0.83	21.21^ab^ ± 0.91	2.17^b^ ± 0.38	1.25^ab^ ± 0.30
**SHOP 6 (BREAST)**	73.52^ab^ ± 0.94	21.09^ab^ ± 0.88	1.97^b^ ± 0.25	1.20^b^ ± 0.32
**Control (THIGH)**	72.21^a^ ± 0.63	19.71^a^ ± 0.86	7.27^a^ ± 0.70	1.06^bc^ ± 0.17
**SHOP 1 (THIGH)**	71.44^b^ ± 0.70	19.13^a^ ± 0.48	7.20^a^ ± 0.59	1.13^ab^ ± 0.14
**SHOP 2 (THIGH)**	71.16^b^ ± 0.80	19.19^a^ ± 0.58	7.43^a^ ± 0.46	1.20^a^ ± 0.16
**SHOP 3 (THIGH)**	71.66^ab^ ± 0.66	19.44^a^ ± 0.77	7.02^a^ ± 0.47	1.11^ab^ ± 0.10
**SHOP 4 (THIGH)**	71.68^ab^ ± 0.51	18.29^b^ ± 0.52	6.93^a^ ± 0.72	0.97^c^ ± 0.14
**SHOP 5 (THIGH)**	71.22^b^ ± 0.73	18.06^b^ ± 0.56	7.35^a^ ± 0.45	0.95^c^ ± 0.12
**SHOP 6 (THIGH)**	71.66^ab^ ± 0.52	18.36^b^ ± 0.76	7.30^a^ ± 0.56	1.08^bc^ ± 0.13

Means with different small letter superscripts (a, b, c, d…) in a column differ significantly (p ≤ 0.05).

#### 3.1.2. Proximate chicken thigh composition

The moisture content of the control thigh sample was significantly higher (*p* < 0.05) than that of the samples from shops 1, 2, and 5. The moisture content of the samples from shops 3, 4, and 6 was statistically comparable to that of the control. The protein content of the thigh samples from the control and those from shops 1, 2, and 3 were significantly higher (*p* < 0.05) than the protein content of the samples from shops 4, 5, and 6, as presented in Table 1. The fat content of the thigh samples did not differ significantly (*p* > 0.05) between the control samples and the samples from the different shops. The ash content of the thigh samples from the control and those from the shops ranged from 0.95 to 1.20%. The ash content of the thigh sample from shop 2 was significantly higher (*p* < 0.05) than that of the control thigh sample and the thigh samples from shops 4, 5, and 6. The ash content of the samples from shop 2 was statistically comparable to that of the samples from shops 1 and 3, as presented in Table 1.

### 3.2. Physico-chemical properties of the chicken meat

#### 3.2.1. Physico-chemical properties of the raw breast muscle sample

The pH of the breast samples from shops 1 and 3 was significantly higher (*p* < 0.05) than that of the control sample and the samples from shops 4 and 5, and it was statistically comparable to that of the sample from shop 2. The WHC of the breast muscle samples from the control and those from the shops ranged from 46.12% to 48.86%. The WHC of the breast muscle samples from shops 1 and 3 was significantly higher (*p* < 0.05) than that of the samples from shops 2, 4, 5, and 6, and the WHC of the breast muscle samples from shops 1 and 3 was statistically comparable to the WHC of the control sample. The WHC of the samples from shops 2, 4, 5, and 6 was significantly lower (*p* < 0.05). The TBA value of the control breast sample was significantly higher (*p* < 0.05) than the TBA value of the sample from shop 4. The TBA value of the samples from shops 1, 2, 3, 5, and 6 was statistically comparable to the TBA value of the control, as presented in Table 2.

**Table 2:** The pH, WHC, and TBA values of the raw chicken breast and thigh muscle samples obtained from the livestock farm, COVS and six different retail outlets across Hisar, Haryana, India (Mean ± SD) (*n* = 6).

**Treatment**	**pH**	**WHC (%)**	**TBA value (mg/ malonaldehyde/ kg)**
**CONTROL (BREAST)**	6.08^c^ ± 0.08	48.11^ab^ ± 1.31	0.16^a^ ± 0.06
**SHOP 1 (BREAST)**	6.19^a^ ± 0.03	48.44^a^ ± 1.86	0.11^ab^ ± 0.08
**SHOP 2 (BREAST)**	6.12^ab^ ± 0.06	47.10^bc^ ± 1.45	0.10^ab^ ± 0.06
**SHOP 3 (BREAST)**	6.21^a^ ± 0.09	48.86^a^ ± 0.97	0.13^ab^ ± 0.1
**SHOP 4 (BREAST)**	6.01^c^ ± 0.07	46.23^c^ ± 1.01	0.09^b^ ± 0.08
**SHOP 5 (BREAST)**	6.04^c^ ± 0.06	46.39^c^ ± 1.26	0.12^ab^ ± 0.03
**SHOP 6 (BREAST)**	6.17^ab^ ± 0.05	46.12^c^ ± 1.60	0.15^ab^ ± 0.05
**Control (THIGH)**	6.20^c^ ± 0.08	54.37^b^ ± 2.22	0.33^a^ ± 0.04
**SHOP 1 (THIGH)**	6.30^a^ ± 0.07	57.18^a^ ± 2.14	0.26^b^ ± 0.08
**SHOP 2 (THIGH)**	6.29^a^ ± 0.02	53.70^bc^ ± 1.31	0.20^c^ ± 0.09
**SHOP 3 (THIGH)**	6.23^bc^ ± 0.05	54.77^b^ ± 1.47	0.21^bc^ ± 0.05
**SHOP 4 (THIGH)**	6.20^c^ ± 0.04	53.97^bc^ ± 1.87	0.32^a^ ± 0.05
**SHOP 5 (THIGH)**	6.27^ab^ ± 0.02	50.85^c^ ± 0.86	0.20^c^ ± 0.07
**SHOP 6 (THIGH)**	6.25^abc^ ± 0.08	51.97^c^ ± 1.75	0.34^a^ ± 0.02

Means with different small letter superscripts (a, b, c, d…) in a column differ significantly (p ≤ 0.05).

#### 3.2.2. Physico-chemical properties of the raw thigh muscle sample

The pH of the samples from shops 1 and 2 were significantly higher (*p* < 0.05) than the pH of the control thigh sample and the samples from shops 3 and 4. Meanwhile, the pH of the samples from shops 5 and 6 was statistically comparable to the pH of the samples from shops 1 and 2. The WHC of the control thigh sample and the samples from the shops ranged from 50.85 to 57.18%. The WHC of the sample from shop 1 was significantly higher (*p* < 0.05) than the WHC of the samples from other shops and the control sample. The WHC of the samples from shops 2, 3, 4, 5, and 6 and that of the control was lower than the WHC of the sample from shop 1. The TBA values of the control thigh sample and the samples from the shops ranged from 0.20 to 0.34 mg/malonaldehyde/kg. The TBA values of the control thigh sample and the samples from shops 4 and 6 were significantly higher (*p* < 0.05) than those of the samples from shops 1, 2, 3, and 5, as presented in Table 2.

### 3.3. Physico-chemical properties of the chicken meat sample

#### 3.3.1. Instrumental color analysis of the raw breast muscle of the chicken

The L^*^ values of the control breast sample and the breast samples of the shops ranged from 49.28 to 57.09. The samples from shops 2 and 3 had the highest lightness (*p* < 0.05) or the lowest darkness compared to the control sample and the samples from shops 1, 4, 5, and 6. The a^*^ values of the control sample and the samples from the shops ranged from 1.92 to 2.78. The breast muscle sample from shop 1 had a significantly higher redness (*p* < 0.05) value compared to the other samples and the control, and the values of the samples from shops 3, 4, 5, and 6 were statistically comparable to that of the sample from shop 2. The b^*^ values of the breast samples from the control and those from the various shops ranged from 4.52 to 6.69. The sample from shop 2 had a significantly higher yellowness (*p* < 0.05) value than the control sample and other samples, as presented in Table 3.

**Table 3:** Instrumental color of raw chicken breast and thigh muscle samples obtained from the livestock farm, COVS and six different retail outlets across Hisar, Haryana, India (Mean ± SD) (*n* = 6).

**Treatment**	L^*^(lightness)	a^*^(redness)	b^*^(yellowness)
**CONTROL (BREAST)**	53.46^b^ ± 2.03	1.94^c^ ± 0.34	4.98^c^ ± 0.65
**SHOP 1 (BREAST)**	53.80^b^ ± 1.32	2.78^a^ ± 0.61	5.23^bc^ ± 0.85
**SHOP 2 (BREAST)**	56.96^a^ ± 2.04	2.72^ab^ ± 0.83	6.69^a^ ± 1.69
**SHOP 3 (BREAST)**	57.09^a^ ± 2.46	1.92^c^ ± 0.61	5.14^c^ ± 0.87
**SHOP 4 (BREAST)**	49.28^c^ ± 2.59	2.33^bc^ ± 0.69	5.45^bc^ ± 1.14
**SHOP 5 (BREAST)**	49.89^c^ ± 2.01	2.16^c^ ± 0.86	4.52^c^ ± 0.45
**SHOP 6 (BREAST)**	49.40^c^ ± 1.01	1.97^c^ ± 0.77	5.83^b^ ± 0.66
**Control (THIGH)**	50.56^a^ ± 2.43	3.39^bc^ ± 1.73	6.28^a^ ± 1.63
**SHOP 1 (THIGH)**	49.56^b^ ± 1.25	3.69^b^ ± 1.36	4.06^c^ ± 0.62
**SHOP 2 (THIGH)**	48.68^b^ ± 1.38	4.02^ab^ ± 0.90	3.33^c^ ± 0.73
**SHOP 3 (THIGH)**	50.71^a^ ± 0.94	4.64^a^ ± 1.32	5.10^b^ ± 1.29
**SHOP 4 (THIGH)**	48.88^b^ ± 1.20	3.65^b^ ± 1.75	3.92^c^ ± 1.20
**SHOP 5 (THIGH)**	49.00^b^ ± 1.10	2.65^c^ ± 0.90	3.73^c^ ± 1.75
**SHOP 6 (THIGH)**	49.40^b^ ± 1.00	2.61^c^ ± 1.03	3.71^c^ ± 1.33

Means with different small letter superscripts (a, b, c, d…) in a column differ significantly (p ≤ 0.05).

#### 3.3.2. Instrumental color analysis of the raw thigh muscle of chicken

The L^*^ values of the thigh sample from the control and that from shop 3 had significantly higher lightness (*p* < 0.05) than the L^*^ values of the samples from shops 1, 2, 4, 5, and 6. The samples from shops 1, 2, 4, 5, and 6 were statistically comparable among themselves. The a^*^ values of the control thigh sample and the samples obtained from various shops varied between 2.61 and 4.64. The sample from shop 3 had a significantly higher redness value than the control and other samples. The b^*^ values of the control thigh sample and those obtained from various shops ranged between 3.33 and 6.28. The control sample had a significantly higher yellowness value compared to the other samples, as presented in Table 3.

### 3.4. Sensory evaluation

The overall acceptability scores of the control breast sample and those from the shops ranged from 3.98 to 4.42. The overall acceptability score of the breast samples from shops 1 and 3 was significantly higher (*p* < 0.05) than the control breast sample and those from shops 2, 4, 5, and 6. The overall acceptability scores of the control breast muscle sample and those from shops 2, 3, 4, 5, and 6 were statistically comparable, as presented in Table 4. The overall acceptability score of the thigh samples from shops 1 and 3 was significantly higher (*p* < 0.05) than those sample from the control and the samples from shops 2, 4, 5, and 6.

**Table 4:** Sensory quality of raw chicken breast and thigh muscle samples obtained from the Livestock Farm, COVS, and six different retail outlets across Hisar, Haryana, India (Mean ± SD) (*n* = 6).

**Treatment**	**Color**	**Aroma**	**General appearance**	**Overall acceptability**
**CONTROL (BREAST)**	4.88^a^ ± 0.23	4.33^ab^ ± 0.49	4.25^b^ ± 0.62	4.01^b^ ± 0.30
**SHOP 1 (BREAST)**	4.13^b^ ± 0.48	4.17^b^ ± 0.25	4.88^a^ ± 0.23	4.42^a^ ± 0.36
**SHOP 2 (BREAST)**	3.83^bc^ ± 0.26	4.04^b^ ± 0.40	4.08^b^ ± 0.47	4.04^b^ ± 0.33
**SHOP 3 (BREAST)**	4.58^a^ ± 0.36	4.63^a^ ± 0.38	4.08^b^ ± 0.36	4.29^a^ ± 0.40
**SHOP 4 (BREAST)**	3.71^c^ ± 0.50	3.33^c^ ± 0.33	3.61^c^ ± 0.43	3.98^b^ ± 0.33
**SHOP 5 (BREAST)**	3.75^c^ ± 0.45	3.46^c^ ± 0.26	3.54^c^ ± 0.50	4.00^b^ ± 0.42
**SHOP 6 (BREAST)**	4.04^bc^ ± 0.26	4.08^b^ ± 0.36	3.96^b^ ± 0.40	4.11^b^ ± 0.45
**Control (THIGH)**	4.46^b^ ± 0.33	4.33^ab^ ± 0.25	4.25^a^ ± 0.50	4.00^b^ ± 0.48
**SHOP 1 (THIGH)**	4.47^b^ ± 0.25	4.13^b^ ± 0.31	4.42^a^ ± 0.56	4.54^a^ ± 0.33
**SHOP 2 (THIGH)**	4.35^b^ ± 0.34	4.01^b^ ± 0.29	4.58^a^ ± 0.60	4.17^b^ ± 0.33
**SHOP 3 (THIGH)**	4.75^a^ ± 0.26	4.54^a^ ± 0.40	4.29^a^ ± 0.33	4.55^a^ ± 0.40
**SHOP 4 (THIGH)**	4.17^c^ ± 0.40	3.54^c^ ± 0.40	3.13^b^ ± 0.43	3.48^c^ ± 0.43
**SHOP 5 (THIGH)**	4.09^c^ ± 0.26	3.67^c^ ± 0.50	3.42^b^ ± 0.56	3.78^bc^ ± 0.25
**SHOP 6 (THIGH)**	4.11^c^ ± 0.43	4.13^b^ ± 0.57	3.29^b^ ± 0.58	3.67^c^ ± 0.44

Means with different small letter superscripts (a, b, c, d…) in a column differ significantly (p ≤ 0.05).

### 3.5. Microbiological evaluation

The coliform count of the breast samples from shops 4, 5, and 6 was significantly higher than the coliform counts of the control sample and the samples from shops 1, 2, and 3 (*p* < 0.05). The total plate count of the breast sample from shop 5 was significantly higher (*p* < 0.05) than those of the control sample and the samples from shops 1, 2, 3, and 6, and the total plate count of the breast sample from shop 5 was statistically comparable to that of the sample from shop 4. The psychrotrophic count of the breast samples from shops 4 and 5 was significantly higher (*p* < 0.05) than of the sample from the control sample and the samples from shops 1 and 2, and the psychrotrophic count of the breast samples from shops 4 and 5 was statistically comparable to that of the sample from shop 6, as presented in Table 5.

**Table 5:** Coliform count, total plate count, and psychrotrophic count (log cfu/g) of raw chicken breast and thigh muscle samples obtained from the Livestock Farm, COVS and six different retail outlets across Hisar, Haryana, India (Mean ± SD) (*n* = 6).

**Treatment**	**Coliform count**	**Total plate count**	**Psychrotrophic count**
**CONTROL (BREAST)**	1.46^c^ ± 0.24	3.93^c^ ± 0.75	1.14^c^ ± 0.61
**SHOP 1 (BREAST)**	1.58^c^ ± 0.31	3.98^c^ ± 0.27	1.30^c^ ± 0.47
**SHOP 2 (BREAST)**	1.49^c^ ± 0.41	3.52^c^ ± 0.52	1.93^b^ ± 0.54
**SHOP 3 (BREAST)**	2.42^b^ ± 0.50	4.77^b^ ± 0.25	1.22^c^ ± 0.37
**SHOP 4 (BREAST)**	3.18^a^ ± 0.32	5.19^ab^ ± 0.59	2.47^a^ ± 0.43
**SHOP 5 (BREAST)**	3.26^a^ ± 0.36	5.28^a^ ± 0.34	2.38^a^ ± 0.40
**SHOP 6 (BREAST)**	3.08^a^ ± 0.44	4.80^b^ ± 0.68	2.21^ab^ ± 0.38
**Control (THIGH)**	1.14^c^ ± 0.26	2.42^c^ ± 0.57	1.07^c^ ± 0.34
**SHOP 1 (THIGH)**	1.20^c^ ± 0.33	2.72^c^ ± 0.53	1.36^c^ ± 0.62
**SHOP 2 (THIGH)**	1.10^c^ ± 0.39	3.07^c^ ± 0.31	1.18^c^ ± 0.32
**SHOP 3 (THIGH)**	1.96^b^ ± 0.49	4.20^b^ ± 0.56	1.72^b^ ± 0.49
**SHOP 4 (THIGH)**	2.64^a^ ± 0.47	5.06^b^ ± 0.66	2.20^a^ ± 0.28
**SHOP 5 (THIGH)**	2.41^ab^ ± 0.35	5.10^a^ ± 0.64	2.53^a^ ± 0.41
**SHOP 6 (THIGH)**	2.70^a^ ± 0.57	4.27^b^ ± 0.53	1.74^b^ ± 0.34

Means with different small letter superscripts (a, b, c, d…) in a column differ significantly (p ≤ 0.05).

The coliform count of the thigh samples from shops 4 and 6 was significantly higher (*p* < 0.05) than those of the control sample and the samples from shops 1, 2, and 3, and the coliform count of the thigh samples from shops 4 and 6 was statistically comparable to that of the sample from shop 5. The total plate count of the thigh sample from shop 5 was significantly higher (*p* < 0.05) than those of the control sample and the samples from all other shops. The psychrotrophic count of the thigh samples from shops 4 and 5 was significantly higher (*p* < 0.05) than those of the control sample and the samples from shops 1, 2, 3, and 6, as presented in Table 5.

## 4. Discussion

### 4.1. Proximate composition of raw chicken meat

There is a reverse relationship between the fat and moisture contents of the breast muscle. A higher proportion of moisture in lean meat makes it easy to cook and digest. The low fat percentage and the higher protein percentage in meat are desirable traits that are preferred by consumers and have better health implications. Chicken breast meat is perceived to be a lean, low fat, and high-protein food (16). The proximate composition of the carcass generally depends on different factors such as diet, age at the time of slaughter, antemortem management, environmental conditions, and genetic makeup (17). Da Silva et al. (18) reported a higher protein value (18.00%) of the thigh cuts of free-range broiler meat, whereas the thigh and drumstick cuts of industrial broiler meat had a higher total fat value. Fakolade (19) reported that the crude protein content of chicken breast meat was significantly higher at 8 weeks of age and significantly lower at 4 weeks of age; the researcher also obtained the least crude protein content of the thigh muscle at 4 weeks of age. Bowker (20) observed in their study that low muscle pH is the primary determinant of myosin denaturation and low WHC in pale soft exudate (PSE)-like broiler breast meat.

Breed hybridization also increases protein, moisture, and ash contents when compared with naturally occurring breeds (21). The stunning or slaughtering techniques also lead to variations in the proximate principles of raw meat. Bostami et al. (22) observed that the proximate compositions of broiler chicken breast (*Pectoralis major*) and thigh (*Flexor cruris medialis*) muscles were comparable among other birds that were subjected to halal neck cutting after no stunning or electrical stunning.

### 4.2. pH of raw chicken breast and thigh muscles

The pH value of the breast sample ranged from 6.01 to 6.21 in the current study. The thigh samples from shops 1, 2, 5, and 6 had significantly higher pH values than the control thigh sample. 21 reported that the chicken breed did not have any effect on the pH values of raw breast meat (*p* > 0.05). However, old broiler hens (OBH) had significantly higher (*p* < 0.01) pH values than old layer hens (OLH) for thigh meat. Hassanin et al. (23) reported a chicken thigh pH value ranging from 5.65 to 5.84, and a breast pH value ranging from 5.70 to 5.96. An increase in the pH can be attributed to the bird's state of welfare and behavior during slaughtering, especially the movement of the wings (10, 24). Devatkal et al. (25) reported that the rate of decline in the pH value depends on the activity of glycolytic enzymes immediately after death, and the ultimate pH was determined by the initial glycogen reserves of the muscle.

### 4.3. The WHC of raw chicken breast and thigh muscles

The WHC of meat is determined by the affinity between the water molecules, myofibrillar protein, and the space retained by water within the myofibril. The decline in the pH value near the isoelectric point of the myofibrillar protein results in a low WHC due to a reduction in the net charge of the myofibrillar protein, which decreases both the water affinity and space thereafter (26). Ghimire and Parajuli (27) reported the WHC values of the thighs, drumsticks, wings, breasts, and torsos as 0.58, 0.41, 0.46, 0.50, and 0.48, respectively. Drumstick meat had a significantly low WHC and thigh meat had a significantly high WHC when compared with other cut-up parts. Bowker (20) observed that low muscle pH is the primary determinant of low WHC in the PSE, such as broiler breast meat.

### 4.4. TBA value of raw chicken breast and thigh muscles

The normal TBA values of chicken breast and thigh meat reported in a previous study are approximately 0.17 and 0.26, respectively (28), and the same values were reported in the current study. Oxidative rancidity of fat in the muscle tissue is determined by its TBA value. The TBA value of a sample shows a steady increase as it becomes more rancid, but less variation is expected between the TBA values in a fresh, raw sample (29). Hassanin et al. (23) found that the TBA values (mg%) of raw chicken breast ranged from 0.06 to 0.14 mg% and that of the thigh ranged from 0.08 to 0.21 mg%.

### 4.5. Instrumental color analysis of raw chicken breast and thigh muscles

The myoglobin content in the muscles, which varies depending on several factors such as the species, age, gender, breed, and pH of the muscle at the time of slaughter, determines the color of the meat. These factors influence the reflectance or transmittance of light across the muscle fiber, which causes lightness and darkness in the meat (30). Ullengala et al. (31) reported that the lightness (L^*^) did not vary significantly among crosses, whereas yellowness (b^*^) and redness (a^*^) varied significantly, which indicates the presence of variable myoglobin content in the muscles. Tyasi et al. (32) observed that the meat color of the breast and thigh of the Ovambo chicken breed had high L^*^ and b^*^ but low a^*^ values. The Potchefstroom Koekoek chicken breed had higher values for all color indicators than other chicken breeds. Lakshani et al. (33) reported that the brighter color of the breast muscle of the spent hen could be attributed to the relatively low pH of its meat.

### 4.6. Sensory quality of raw chicken breast and thigh muscles

The sensory evaluation scores are influenced by many factors, such as genetics, breed, age, gender, rearing practices, and scientific feeding. Çapan and Bagdatli (34) studied that the general appearance of conventionally grown chicken breast, the odor score, and the overall acceptability score of the conventionally grown chicken thigh were highly appreciated by the panelists compared to that of organic chicken. Uddin (35) reported that there was no significant difference (*p* < 0.05) in the sensory evaluation of non-descriptive native and naked-neck chicken meat. Indigenous chicken meat obtained from hens had better scores than that obtained from their male counterpart. Indigenous broilers also obtained higher sensory evaluation scores than commercial broilers.

### 4.7. Microbiological status of raw chicken breast and thigh muscles

The control thigh sample had a significantly lower total plate and psychrotrophic count than the samples from shops 3, 4, 5, and 6. Nurmasytha et al. (36) reported that the TPC of a market sample of raw chicken breast meat ranged from 2.5 × 10^5^ to 1.9 × 10^6^ CFU/g, and *E. coli* in raw chicken breast meat ranged from 1.6 × 10^3^ to 3.0 × 10^4^ CFU/g. In a study by Mohammed (37), fresh chicken thigh samples were found to be more contaminated according to the mean values of the APC and *Enterobacteriaceae*. Hassanin et al. (38) reported that the psychrotrophic counts of wing and breast samples were 4.91 × 10^5^ and 3.88 × 10^5^ CFU/g, respectively, which indicated that the wings were the most contaminated samples and that breast samples had the lowest contamination. Fresh chicken meat may be contaminated with different food pathogens due to personal unhygienic culpabilities that occur during various slaughter, storage, transportation, and handling processes, including contaminated water, gastrointestinal contamination, and air, dust, sewage, and environmental surfaces (39). Hence, the hygienic practices performed during slaughtering and processing, the storage temperature and time, and the health status of the slaughtered birds are the determinant factors for the bacteriological profile of fresh raw poultry meat.

## 5. Limitations

A potential limitation of this study is its cross-sectional design. Deeper insights about the raw meat status in Hisar, Haryana, India can only be drawn by planning a comprehensive and round-the-year study that covers a larger area and includes more shops and an even higher number of quality indicators of fresh meat. This study can be classified as a preliminary step in this direction. There are no refrigeration facilities in the meat retail shops, due to which some shops have poor microbiological parameters. Sanitation practices are also poor, and no disinfectants or sanitizers are used.

## 6. Conclusions

This study concluded that the meat obtained by slaughtering animals under the supervision of trained professionals and laboratory conditions has better hygienic quality, which is indicated by the lower microbial count and other physico-chemical parameters. The meat samples collected from shops in this study varied significantly, which could have been due to the different hygienic practices being followed at the retail level. The meat retail environment needs to be improved, and various measures, such as educating and training butchers regarding hygienic meat production, must be taken into account. This would enable safe meat production with better price returns for consumers. Policymakers should primarily aim to bridge the gap between laboratory conditions and real-time retail conditions in terms of hygienic mandates.

## Data availability statement

The datasets that were generated during and/or analyzed while the current study was conducted are available from the corresponding author upon request.

## Ethics statement

Ethical review and approval was not required for the study on human participants in accordance with the local legislation and institutional requirements.

## Author contributions

This work was conducted and written: IB, AM, AR, SS, SY, RZ, LL, HH, and NT. Funding acquisition: LL, HH, and AR. Project administration: AR. Writing—review and editing: AR and NT. All authors have read and agreed to the published version of the manuscript.
