# Supplementary material for: Physico-chemical, sensory, and microbiological quality of raw chicken meat: an exploratory study in the Hisar city of Haryana, India
Source: Front Nutr. 2023 Jul 18;10:1184005. doi: 10.3389/fnut.2023.1184005 (PMC10391170; doi:10.3389/fnut.2023.1184005)
Supplement: Supplementary file 1 [file Data_Sheet_1.pdf]

APPENDIX –I

Details of the six shops used in the study

Shop no.	Shop size	Daily turn over	Shop address	Meat storage facility	Meat packaging facility
S1	Small	30 kg/day	Professor Colony, Hisar, Haryana, India	Present	Present
S2	Small	20 kg/day	Chandan Nagar, Hisar, Haryana, India	Absent	Present
S3	Small	20 kg/day	Azad Nagar, Hisar, Haryana, India	Absent	Present
S4	Small	30 kg/day	Patel Nagar, Hisar, Haryana, India	Absent	Present
S5	Large	150 kg/day	Thandisadak, Hisar, Haryana, India	Present	Present
S6	Medium	60-70 kg/day	Mahavir colony, Hisar, Haryana, India	Present	Present

APPENDIX -II

Department of Livestock Products Technology

Lala Lajpat Rai University of Veterinary and Animal Sciences, Hisar, Haryana, India

Proforma for Sensory evaluation

Panelist's name: **Sample's name:** **Date:**

Sensory attributes	Descriptive scale for sensory attributes of raw chicken meat				
	5	4	3	2	1
**Color**	Characteristic red color	Acceptable color	Moderately appealing	Slightly appealing	Unappealing
**Aroma**	Highly desirable	Desirable	Moderately appealing	Slightly appealing	Unappealing
**General appearance**	Highly desirable	Desirable	Moderately desirable	Slightly desirable	Slightly undesirable
**Acceptability**	Very much acceptable	Acceptable	Moderately Acceptable	Slightly acceptable	Unacceptable

Sample	Color	Aroma	General appearance	Acceptability
1				
2				
3				
4				
5				
6				
7				
8				

Remarks: **Signature**
